# Head tremor in Parkinson´s Disease, clinical Associations and response to therapy

**DOI:** 10.1016/j.prdoa.2025.100328

**Published:** 2025-04-12

**Authors:** José Fidel Baizabal-Carvallo, Marlene Alonso-Juarez, Robert Fekete

**Affiliations:** a Department of Sciences and Engineering, University of Guanajuato, León, México; b Instituto Politécnico Nacional, Mexico; c New York Medical College, Valhalla, NY, USA

**Keywords:** Parkinsońs disease, Tremor, Jaw, Clinical correlations, Head tremor

## Abstract

**Background:**

Tremor is frequently observed in patients with Parkinsońs disease (PD). Tremor most commonly affects the upper limbs but may also affect the axial muscles in PD. Head tremor (HT) is usually identified in patients with essential tremor (ET) and cervical dystonia (CD), but rarely reported in PD.

**Objectives Methods:**

We aimed to assess the frequency, clinical features, correlates, and underlying mechanisms of HT in PD.

**Results:**

Among 229 patients with PD, we identified 19 (8.3 %) of patients with HT. There were 11 males and 8 females with a median age at evaluation of 62.0 years. Five patients had slight, ten had mild and four had moderately severe HT. Yes-yes HT was the most common type. HT was associated with PD in 13 patients, eight of them had severe tremor-dominant presentation. In 3 patients there were signs suggesting underlying ET, while 3 patients had CD. Complete resolution of HT was observed with levodopa and/or deep brain stimulation (DBS) in patients with PD only, but inconsistent improvement was observed with comorbid ET or CD. Longer evolution time since PD onset (*P* = 0.024), rest tremor (*P* = 0.001) and CD (*P* = 0.003) were independently associated with HT in the multivariate analysis.

**Conclusions:**

HT was identified in 8.3% of patients with PD. It associated with longer evolution since PD onset, the severity of rest tremor and presence of CD. HT is observed in the context of PD only, particularly in those with severe tremor-dominant presentation, comorbid ET or CD. Most patients improve with dopaminergic therapy or DBS.

## Highlights

• Head tremor was observed in 8.3% of patients with Parkinsońs disease.
• Head tremor was observed with diverse degrees of Parkinsońs disease severity.
• Head tremor may appear at different times within the course of Parkinsońs disease.
• Comorbid essential tremor and cervical dystonia is observed in some of these patients.
• Head tremor in PD correlates with rest tremor, longer evolution and cervical dystonia.

## 1 Introduction

Parkinsońs disease (PD) is the second most common neurodegenerative disorder, characterized by several motor and non-motor manifestations such as muscle rigidity, bradykinesia, abnormal gait, and tremor. While limb rest tremor is a common manifestation in PD, affecting between 65.4 % and 69.6 % of these patients; tremors involving axial muscles, such as jaw tremor are less commonly observed [1]. Head tremor (HT) has also been reported in patients with PD showing a frequency of 4–6 Hz similar to limb rest tremor [2]. HT is identified in 44.4 % of patients with essential tremor (ET) and 68.4 % of patients with cervical dystonia (CD) [3,4]. However, the frequency, clinical characteristics, and correlations of HT in PD have been rarely studied. We aimed to clarify the features of HT in a cohort of patients with PD.

## 2 Materials and Methods

### 2.1 Study population

We assessed 229 consecutive patients with PD attending a tertiary care clinic for movement disorders. Patients were diagnosed according to the Queen Square Brain Bank criteria [5] and patients of any age and both sexes were enrolled in the study. We excluded patient with ET and isolated parkinsonian features, i.e. rest tremor, bradykinesia or rigidity only (8 cases) not fulfilling diagnostic criteria for PD. Patients fulfilling diagnosis criteria for PD and suspected underlying ET were included in the study. The presence of voice or facial tremor and preferential improvement of action tremor with propranolol (instead of levodopa) were features supporting an underlying ET [1]. The evaluation of the motor features of PD were performed by a movement disorders clinician (JFB-C) by means of the Movement Disorders Society Unified Parkinsońs Disease Rating Scale part III or motor score (MDS-UPDRS-III) [6]. For the purpose of this study, severe motor PD was considered with an MDS-UPDRS-III ≥ 60 in the medication and/or deep brain stimulation (DBS) “off” state. However, to summarize and compare the clinical features of patients with and without HT, composite scores for bradykinesia (items: 3.4–3.8), rigidity (item 3.3 of neck and limbs rigidity), postural tremor (item 3.15 bilateral upper limbs), kinetic tremor (item 3.16 bilateral upper limbs) and rest tremor (item 3.17, four limbs) were used [6]. Patients were evaluated before the next dose of levodopa in case they were taking this medication. Additionally, the functional disability stage was assessed with the modified Hoehn & Yahr [HY] scale. The cognitive evaluation was performed with the Montreal Cognitive Assessment (MoCA).

The clinical features of head tremor were evaluated in the sitting position and lying down at 30°. Tremor severity was classified as, slight: <0.5 cm; mild: 0.5 to < 2.5 cm, moderate: 2.5 to 5 cm and, severe: >5 cm or disfiguring. The tremor axis was classified into “yes-yes”, “no-no”, or “round-round”. We assessed the presence of alternating axes in HT. For patients where the HT was not witnessed by the examiner but endorsed by the patient or family members, the clinical features were also registered. We also evaluated the presence and features of cervical dystonia and of dystonic HT such as the presence of null-point (no HT in a specific posture), sensory tricks or pulling sensation of the head. The antiparkinsonian medications were registered, and the levodopa equivalent daily dose (LEDD) was calculated for each patient [7].

### 2.2 Statistical analysis

Data were summarized in percentages, medians, and interquartile ranges (IQRs)*.* The non-parametric Mann-Whitney *U* test was used to compare continuous variables between groups as the Kolmogorov-Smirnov test was consistent with non-normality distribution of such variables. The *x*^2^ test with Yates’s continuity correction or the Fisheŕs exact test was employed to compare nominal or ordinal data between groups*.* We carried out a multivariate logistic regression analysis with the backward Wald́s method and “presence of head tremor” as the dependent variable to assess the association of variables showing a statistically significant association in the bivariate analysis. Odds ratios (ORs) with 95 % confidence interval (C.I.) of independent variables were calculated using the (exp)^B^ coefficients. Goodness of fit of the regression model was estimated with the Hosmer-Lemeshow test and the Nagelkerke test was used to calculate the determination R^2^ coefficient*.* Calculations were performed using SPSS version 22, a *P* value < 0.05 was considered significant.

## 3 Results

We studied 229 consecutive patients with PD. There were 129 males (56.3 %) and 100 females (43.7 %). Head tremor was identified in 19 (8.3 %) of patients. There were 11 males and 8 females, these patients had a median (IQR) age at evaluation of 62.0 years (58.0, 75.0). According to the tremor severity, n = 5 had slight head tremor, n = 10 had mild, n = 4 moderate and no patient had severe HT. Analysis of tremor axis showed n = 8 patients with “yes-yes” HT, n = 7 with “no-no” HT, n = 2 with complex or alternating axes (round-round and no-no), and in n = 2 patients the tremor axis was undetermined, as HT was not witnessed by the examiner, but endorsed by the patient and/or family member without specifying movement axis. Two patients reported HT along with PD onset, n = 2 within 1 year since PD onset and n = 2 patients reported HT onset long after onset of PD, the onset of HT within the disease course was unclear for n = 13 patients (Table 1).

**Table 1:** Summary of clinical features of patients in this cohort.

**Case**	**Age/Sex**	**Evolution time**	**MDS-UPDRS-III score, HY**	**Clinical features of head tremor (axis, severity)**	**Tremor (T) scores**	**Clinical Correlates**	**Effect of treatment**
**1**	48/M	10 (y)	102 (Off), HY, 5	Alternating axis: round-round to no-no, moderate persistent. *	Jaw 0 Kinetic 6 Postural 6 Rest 12 T Persis 4	Severe PD	HT disappears in the levodopa “On” state
**2**	65/F	30 (y)	100 (Off), HY, 5	Yes-yes, moderate persistent. HT onset > 20 y after PD onset	Jaw 0 Kinetic 8 Postural 8 Rest 14 T Persis 4	Severe PD	HT disappears in the levodopa and DBS “On” state
**3**	54/M	8 (y)	114 (Off), HY, 5	Yes-yes, mild to moderate persistent. *	Jaw 4 Kinetic 8 Postural 8 Rest 16 T Persis 4	Severe PD	HT disappears in the levodopa “On” state
**4**	63/M	16 (Y)	68 (Off), HY, 3	Yes-yes, moderate intermittent. *	Jaw 0 Kinetic 6 Postural 6 Rest 12 T Persis 4	Severe PD	HT disappears in the levodopa “On” state
**5**	70/F	7 (y)	61 (Off), HY	Unclear axis, mild, intermittent. HT since PD onset, then disappeared.	Jaw 0 Kinetic 0 Postural 0 Rest 7 T Persis 3	Severe PD	HT disappears in the levodopa and pramipexole “On” state
**6**	72/F	10 (y)	63 (Off), HY, 3	Alternating axis: round-round to no-no, moderate severity, persistent. HT onset within 1 y after PD onset.	Jaw 3 Kinetic 2 Postural 3 Rest 6 T Persis 4 Lingual T 3+	Severe PD	HT disappears in the levodopa “On” state
**7**	53/F	11 (y)	90 (Off), HY, 5	Yes-yes, moderate intermittent. *	Jaw 0 Kinetic 0 Postural 0 Rest 12 T Persis 3	Severe PD	HT disappears in the levodopa “On” state
**8**	48/M	12 (y)	84 (Off), HY, 4	No-no, mild persistent. *	Jaw 1 Kinetic 0 Postural 4 Rest 8 T Persis 4	Severe PD	HT disappears in the levodopa “On” state
**9**	83/M	3 (y)	37 (Off), HY, 3	No-no, moderate intermittent. *	Jaw 0 Kinetic 0 Postural 2 Rest 4 T Persis 3	None	HT disappears in the levodopa “On” state
**10**	83/M	1 (y)	20 (Off), HY	Unclear axis, mild sporadic (only by history). HT since PD onset, then disappeared.	Jaw 3 Kinetic 0 Postural 2 Rest 3 T Persis 2	None	Unclear effect of rasagiline and levodopa
**11**	62/F	20 (y)	27 (Off), HY, 3	Yes-yes, mild intermittent. HT onset 16 y after PD onset.	Jaw 2 Kinetic 0 Postural 0 Rest 7 T Persis 3	None	HT disappears in the levodopa “On” state
**12**	60/M	4 (y)	20 (Off), HY, 3	No-No, moderate, intermittent. *	Jaw 0 Kinetic 2 Postural 0 Rest 0 T Persis 0	None	HT disappears in the levodopa “On” state
**13**	63/F	2 (y)	26 (Off), HY, 2	No-no, moderate intermittent. *	Jaw 0 Kinetic 4 Postural 4 Rest 2 T Persis 4	None	HT disappears in the levodopa “On” state
**14**	83/M	25 (Y)	43 (Off), HY, 3	Yes-yes, moderate intermittent. *	Jaw 0 Kinetic 4 Postural 5 Rest 4 T Persis 1	Probable associated ET.	Improvement with propranolol only
**15**	87/F	45 (Y)	65 (Off), HY, 4	Yes-yes, moderate intermittent. *	Jaw 2 kinetic 5 Postural 4 Rest 4 T Persis 2	Probable associated ET.	HT did not improve with levodopa
**16**	74/F	11 (y)	38 (Off), HY, 4	No-no, moderate sporadic (only by history). HT onset within 1 y after PD onset.	Jaw 2 Kinetic 4 Postural 4 Rest 6 T Persis 4 Voice T 1 + Facial T 3+	Probable associated ET.	HT partial improvement with levodopa, additional improvement with propranolol
**17**	58/F	9 (y)	36 (Off), HY, 3	Yes-yes, moderate intermittent. *	Jaw 0 Kinetic 4 Postural 7 Rest 12 T Persis 2	CD with right shoulder elevation + mild left laterocollis	HT improved with levodopa
**18**	75/M	2 (y)	31 (Off), HY, 3	No-no, moderate intermittent. *	Jaw 0 Kinetic 0 Postural 2 Rest 0 T Persis 0	Dystonic tremor, mild torticollis to the left	Partial improvement with levodopa
**19**	60/M	3 (y)	44 (Off), HY, 3	No-no, moderate intermittent. *	Jaw 3 Kinetic 0 Postural 0 Rest 5 T Persis 4	CD with mild laterocollis to the left	Mild improvement with levodopa

CD: Cervical dystonia; F: Female; HY: modified Hoehn-Yahr scale; M: male; PD: Parkinsońs disease; Persis: persistence; T: tremor; y: years. * Unclear onset within the disease course.

When examining the underlying mechanisms, HT was merely attributed to PD in 13 instances (cases, 1 to 13), under the arguments of prominent response to levodopa, dopaminergic agonists, or bilateral deep brain stimulation (DBS) in the subthalamic nucleus (STN) in all cases and lack of another disorder explaining the HT. Among these patients, there were 8 patients (cases, 1 to 8) with severe motor symptoms in the medication “off” state (MDS-UPDRS-III ≥ 60 points) all of them displayed moderate to severe rest tremor in the upper limbs and other body parts. Patients with severe parkinsonism frequently (6 out of 8) had persistent (or continuous) HT in the medication “off” state, while HT was intermittent in those without severe motor symptoms (Table 1).

There were signs consistent with underlying ET in 3 patients (cases 14 to 16). This assumption was based on the presence of moderate to severe postural and/or kinetic upper limb tremor with incomplete clinical response of HT by dopaminergic therapy, including levodopa, but moderate improvement with propranolol (cases 14 and 16) (Table 1). Moreover, tremor improved when lying down in two patients (cases 14 and 16) supporting the notion of underlying ET. There were 3 additional patients (cases 17 to 19) with signs of CD. These patients showed incomplete response to levodopa. Two patients with CD showed null-point, but no patient endorsed a clear head pulling sensation or showed sensory tricks. These features along with the cervical dystonic postures suggest a role of CD in the pathogenesis of HT in these patients.

When comparing patients with HT with the rest of the cohort, there were no differences in sex distribution, or age at evaluation; however, patients with HT had a longer evolution time (*P* = 0.010) (Table 2). The severity of most motor features did not differ between patients with and without HT; however, patients with HT had higher composite scores in bradykinesia, postural, kinetic and rest tremor, as well as in jaw tremor and rest tremor persistence (*P* < 0.05 for all comparisons). The total MDS-UPDRS-III score was also higher in patients with HT (*P* = 0.008), nonetheless, the proportion of antiparkinsonian medications and LEDD did not differ between groups (Table 2). CD was more commonly identified in patients with HT (OR: 12.94, 95 % CI: 2.41–69.36, *P* = 0.008). No differences were observed for cognitive status or the presence of dyskinesia (OR: 1.66, 95 % CI: 0.52–5.37, *P* = 0.491). In the multivariate analysis, evolution time (*P* = 0.024), comorbid CD (*P* = 0.003), postural (*P* = 0.095) and rest (*P* = 0.001) tremor were independently associated with HT and included in the final regression model (supplementary table).

**Table 2:** Contrasting features between Parkinsońs disease patients with and without head tremor.

	**Head tremor** **Presence** **n = 19 (%)**	**Head tremor** **Absence** **n = 210 (%)**	***P*-value**
Sex (male)	11 (57.9)	118 (56.2)	1.000
Age (years)	62.0 (58.0, 75.0)	69.5 (59.25, 75.0)	0.482
Evolution time (years)	4.0 (2.0, 11.0)	4.0 (1.62, 8.0)	**0.010**
**MDS-UPDRS-III *subscores***			
Language	1.0 (0.0, 2.0)	1.0 (0.0, 2.0)	0.322
Facial expression	1.0 (1.0, 2.0)	1.0 (0.0, 2.0)	0.164
Rigidity (composite score)	9.0 (5.0, 15.0)	7.0 (4.0, 10.0)	0.087
Bradykinesia (composite score)	20.0 (7.0, 32.0)	12.0 (6.0, 19.0)	**0.036**
Rising from chair	1.0 (0.0, 3.0)	1.0 (0.0, 2.0)	0.641
Gait	2.0 (1.0, 3.0)	2.0 (1.0, 3.0)	0.410
Freezing	0.0 (0.0, 3.0)	0.0 (0.0, 1.0)	0.280
Postural stability	2.0 (1.0, 3.0)	2.0 (1.0, 2.0)	0.218
Posture	1.0 (1.0, 2.0)	1.0 (0.0, 2.0)	0.642
Postural tremor (composite score)	4.0 (0.0, 6.0)	0.0 (0.0, 2.0)	**<0.001**
Action tremor (composite score)	2.0 (0.0, 5.0)	1.0 (0.0, 2.0)	**0.015**
Jaw tremor	0.0 (0.0, 2.0)	0.0 (0.0, 1.0)	**0.001**
Upper limb rest tremor (bilateral score)	4.0 (3.0, 6.0)	1.0 (0.0, 3.0)	**<0.001**
Rest tremor (composite score)	6.0 (4.0, 12.0)	2.0 (0.0, 4.0)	**<0.001**
Tremor persistence	3.0 (2.0, 4.0)	1.0 (0.0, 3.0)	**<0.001**
**Total score**	44.0 (31.0, 84.0)	32.0 (23.0, 47.5)	**0.008**
**Hoehn-Yahr scale** (modified)	3.0 (3.0, 5.0)	3.0 (3.0, 4.0)	0.088
Cervical dystonia	3 (15.8)	3 (1.42)	**0.008**
Dyskinesia presence	4 (21.1)	29 (13.8)	0.491
Cognitive status (MoCA)	23.0 (19.0, 25.0)	23.0 (18.0, 26.0)	0.830
**Treatment**			
Levodopa/carbidopa	13 (68.4)	126 (60.0)	0.635
Amantadine	4 (21.1)	16 (7.6)	0.069
Dopamine agonists	4 (21.1)	59 (28.1)	0.696
Anticholinergic	1 (5.3)	16 (7.6)	1.000
MAOI	3 (15.8)	20 (9.5)	0.417
**LEDD**	550.0 (0.0, 975.0)	470.0 (0.0, 950.0)	0.656

Limb bradykinesia composite score (range: 0–40); rigidity composite score (range: 0–20); postural tremor composite score (range: 0–8); kinetic tremor composite score (range: 0–8); limb rest tremor composite score (range: 0–16). LEDD: levodopa equivalent daily dose; MAOI: monoamine oxidase inhibitor; MDS-UPDRS: Movement Disorders Society-Unified Parkinsońs Disease Rating Scale; MoCA: Montreal Cognitive Assessment.

## 4 Discussion

In this study, we identified 19 (8.3 %) cases with HT in a cohort of 229 patients with PD. HT is classically considered uncommon in patients with PD; however, one study enrolling 81 patients with PD reported HT in 17 % of them [8]. The frequency of HT may be related to the ascertainment of the cases. In our study, patients were interrogated and examined for the presence of HT, increasing the probability of a correct determination in the frequency of HT in PD.

While HT is 4 to 6 times more common in women with ET [9], we did not find a difference in sex distribution for PD patients with and without HT. No difference in age was observed, but patients with HT had a longer evolution time of PD and were more severely affected than those without HT. The latter suggests that HT in PD tends to appear within the course of the disease, sometimes coinciding with the development of more severe rest limb tremor; however, HT onset early into the disease course may be observed in some cases. HT was independently associated with the composite rest tremor score in the multivariate analysis, and patients with HT associated with PD showed a clear benefit with levodopa and/or bilateral DBS in the STN. These observations support the notion that HT in PD shares pathophysiology mechanisms with jaw and limb rest tremor, as suggested by another study of 5 patients with PD and HT [2].

Another group of patients had probable associated ET, this was supported by the presence of voice tremor, facial tremor plus moderate to severe upper limb kinetic tremor and HT preferentially improving with propranolol rather than levodopa. While the coexistent of ET and PD is recognized to occur in some patients [10], the diagnosis of these patients may be challenging particularly when they do not have a clear history of ET preceding the onset of PD; moreover, some patients with ET may have parkinsonian features not fulfilling a clear diagnosis of PD [10].

Finally, a third group of 3 patients had HT in the context of CD, two of them had features of dystonic tremor. While HT may be a manifestation of CD as pointed out by Troung and Hermanowicz [11]. These patients had an inconsistent response with levodopa, but CD was independently associated with HT in our cohort, by controlling for other variables in the multivariate regression analysis (*P* = 0.003). Comorbid CD has also been associated with HT in patients with ET [3]. But the presence of ET plus CD was not observed in any of our patients. While tremor is observed in 53.3 % of patients with different types of isolated dystonia [12]. Several forms of dystonia have been described in patients with PD with a prevalence up to 30 % [13]. CD has been reported as the initial symptom in 2.4 % of patients with PD, sometimes preceding the onset of motor symptoms. Patients with dystonia may show an inconsistent response to levodopa with some cases showing exacerbation of dystonia with dopaminergic medication [14]. But this phenomenon was not observed in our study.

The pathophysiology of tremor in PD is complex. Evidence has pointed into a dimmer-switch model, where the basal ganglia initiate a tremor episode (switch) and the cerebello-thalamo-cortical (CTC) circuit modulates tremor amplitude (dimmer) [15]. It has been demonstrated that dysfunction of the CTC circuit also occurs in patients with ET and dystonia [16]. However, such dysfunction may have fundamental neurophysiological differences among disorders. For example, functional connectivity of the CTC circuit seems to be decreased in patients with dystonia compared with other disorders [16]. Moreover, a group of patients with PD has been identified with levodopa-resistant tremor. In those cases, dopamine may be less effective reducing tremor-related activity in the cerebellar thalamus and increased activity in non-dopaminergic regions such as the cerebellum [15]. It is unclear whether this occurs in patients with pure PD and HT, but it is more likely to occur when comorbid ET and/or CD are present [15].

Our study has limitations, as patients were not assessed with neurophysiology studies to estimate the frequency and severity of head tremor. We estimated the time frequency of HT between 4 to 6 Hz for most of our patients; however, we did not assess this variable by formal neurophysiology. This study was conducted in a single reference center for PD, potentially affecting generalizability of results; however, all patients were evaluated by a single physician and the severity range, evolution time and demographic features of the cohort was considered representative for patients with this disorder. Further studies should compare patients with PD and HT with ET or CD with HT to address specific clinical features for each disorder.

## 5 Conclusions

Head tremor was identified in 8.3 % of PD patients. HT may present in the context of PD only, with a proportion of patients having severe tremor-dominant PD, other patients showed signs of comorbid ET and CD, suggesting that more than single pathophysiology underlies HT observed in patients with PD. Patients with PD and HT had longer evolution time and greater motor impairment with more severe rest tremor. HT readily improves with levodopa when no other disorder is identified besides PD, but it has an inconsistent response to levodopa when associated with ET or CD.

## CRediT authorship contribution statement

**José Fidel Baizabal-Carvallo:** Writing – review & editing, Writing – original draft, Visualization, Validation, Resources, Methodology, Investigation, Formal analysis, Conceptualization. **Marlene Alonso-Juarez:** Writing – review & editing, Methodology, Investigation, Formal analysis, Data curation. **Robert Fekete:** Writing – review & editing, Visualization, Validation, Resources.

## Declaration of competing interest

The authors declare that they have no known competing financial interests or personal relationships that could have appeared to influence the work reported in this paper.
