# Supplementary material for: Head tremor in Parkinson´s Disease, clinical Associations and response to therapy
Source: Clin Park Relat Disord. 2025 Apr 12;12:100328. doi: 10.1016/j.prdoa.2025.100328 (PMC12020866; doi:10.1016/j.prdoa.2025.100328)
Supplement: Supplementary Data 1 [file mmc1.docx]

**Supplementary table. Multivariate analysis of statistically significant variables in the bivariate analysis**.

| **Variables in the final equation** | **B coefficient** | **Standard error** | **Wald** | ***P*-value** | **OR (Exp^B^)** | **Exp^B^ 95% C.I.** |
| --- | --- | --- | --- | --- | --- | --- |
| Evolution time  Postural tremor *  Rest tremor *  Cervical dystonia | 0.075  0.303  0.309  3.095 | 0.033  0.181  0.095  1.047 | 5.066  2.794  10.545  8.739 | **0.024**  **0.095**  **0.001**  **0.003** | 1.078  1.354  1.363  22.093 | 1.010-1.151  0.949-1.931  1.130-1.642  2.838-171.99 |

Constant: B-5.098, *P*<0.001. Hosmer-Lemeshow: (*P*=0.594). Nagelkerke R^2^=0.436. Variables not included in the final equation: jaw tremor severity, bradykinesia composite score, kinetic tremor, tremor persistence, total MDS-UPDRS-III score. * Composite scores.
